# Preliminary Assessment of Commercial Antibacterial Finishes on Knitted Fabrics from Recycled Cotton and Polyester

**DOI:** 10.3390/ma18235319

**Published:** 2025-11-25

**Authors:** Muhammad Sajid Faheem, Hafsa Jamshaid, Rajesh Kumar Mishra, Adeel Abbas, Miroslav Muller, Jiri Urban, Michal Penc

**Affiliations:** 1 Department of Textiles, School of Engineering & Technology, National Textile University, Faisalabad 37610, Pakistan; sajid.faheem@ntu.edu.pk (M.S.F.); hafsa@ntu.edu.pk (H.J.); 2 Department of Material Science and Manufacturing Technology, Faculty of Engineering, Czech University of Life Sciences Prague, Kamycka 129, Suchdol, 165 00 Prague, Czech Republic; muller@tf.czu.cz (M.M.); urbanjiri@tf.czu.cz (J.U.); penc@tf.czu.cz (M.P.); 3 School of Fashion and Textiles, RMIT University, Melbourne, VIC 3000, Australia

**Keywords:** recycled textiles, metal oxide nanoparticles, antibacterial activity, functional textiles, thermal comfort, serviceability

## Abstract

Recycled materials are employed in several areas to reduce the environmental burden. Therefore, recycling textiles is a vast domain being investigated by several researchers. Functionalization of textiles using active nanomaterials is a growing area of interest nowadays. However, functionalized textiles with antibacterial properties still do not employ the incorporation of recycled materials. The huge consumer need for different types of functional textiles necessitates a focus on recycled textiles in this area. Hence, this research focuses on the development of metal oxide nanoparticle-functionalized antibacterial textiles using recycled cotton and polyester yarns. Two different finishes have been used with a 50:50 ratio. Using a two-fold approach, antibacterial finishes were applied during both the yarn and fabric stages to analyze the differences in performance. Agar plate qualitative antibacterial analysis revealed efficient antibacterial function both before and after commercial laundering. However, thermo-physiological comfort properties were found to be variable for recycled yarn types and finishing stages. The air permeability of fabric-finished specimens was found to be about 47% lower than yarn-finished specimens owing to reduced porosities. However, the overall moisture management capability (OMMC) index was found to be 21% higher than that of yarn-finished specimens. Among serviceability parameters, bursting strength was found to decrease with increasing concentration, as the finishing treatments made the yarns crispier which eventually compromised yarn strength.

## 1. Introduction

Recycling textiles has gained much popularity in recent times owing to its crucial role in decreasing the environmental burden [1]. Manufacturing products using virgin fiber materials increases the environmental burden; hence, different techniques of mechanical and chemical recycling have been introduced into the market [2]. Such techniques enhance the possibilities of the service life of textile materials through reuse in some other products [3]. However, textile fibers usually lose their mechanical strength during recycling which demands the adoption of appropriate fabrication methods that can handle these delicate recycled materials effectively [4]. Knitting technology can convert single yarns into fabric, with lower effort and more convenience [5]. The weaving process involves higher mechanical stresses during the preparatory stage (warping, sizing, and drawing), as well as during manufacturing (shedding, picking, and beating up). Knitting can handle soft, delicate, and low-strength yarns due to its simpler manufacturing processes. Hence, the knitting process is more suitable for recycled yarns. Knitted fabrics are also preferable for their form fitting, stretching and recovery, and thermo-physiological comfort attributes [6]. This is why knitted fabrics are a primary choice for comfortable clothing [7]. However, such recycled yarn knitted fabrics can also potentially be employed in some functional applications where slightly lower mechanical attributes are also acceptable, and functional performance is more dominant over casual clothing characteristics [8]. Antibacterial knitwear is used for resisting bacterial infections caused due to body sweat and can be engineered using recycled materials [9]. The lower number of twists in recycled yarns make them ideal for knitting, and such attributes also work for enhanced absorption of functional finishes and efficient handling of body perspiration to maintain the body heat balance [10]. Moreover, recycled yarns are often made by blending recycled fibers with some virgin materials which also serve to tailor functional and thermo-physiological comfort characteristics.

In recent years, increasing cases of deaths caused by viral infections such as SARS (severe acute respiratory syndrome), norovirus, and avian influenza have been reported [11,12,13,14,15,16,17]. Findings have been reported about the integration of recycled fibers with some functional materials for antibacterial characteristics [18]. Researchers have engineered antibacterial nonwoven felts by incorporating antibacterial properties into recycled polyester using nano zinc oxide during a spun bonding process [19]. Other researchers have worked on different chemical fixation methods for imparting antibacterial properties to recycled polyester nonwoven felts [20]. On the other hand, some researchers have investigated the properties of yarns made from waste polyester bottles and silica aerogels to produce antibacterial knitwear. However, such fabrics have decreased thermal conductivity and enhanced hydrophobicity [21]. Some others have developed recycled polyester and silver nanocomposite (SNC) polyester blended yarns to engineer antibacterial fabrics. Increasing SNC polyester content improved the antibacterial performance, but surface textures and touch characteristics were compromised [22].

The above-mentioned techniques are viable to design functional textiles with recycled materials. These methods involve chemical recycling and the aforementioned processing methods. However, there is still a huge amount of mechanically recycled yarn being produced on an industrial scale, and such yarns also require some fruitful directions of employment in their second service lives. Scientific studies on these materials are limited to certain conventional attributes. Researchers performed a comparative analysis of the thermal comfort attributes of knitted fabrics engineered using different blends of recycled cotton and polyester [23]. It has been found that the thermo-physiological comfort properties of recycled cotton/polyester knitted fabrics are comparable to those of virgin materials, proposing the use of recycled fibers in knitwear [24]. Another analysis proved that the bursting strength of recycled polyester-based knitwear is equivalent to that of virgin polyester knitted fabric [25]. However, 2.3% higher microfiber leaching from recycled polyester knitted fabrics was observed [26]. Similarly, several researchers have focused on comparisons of the thermal comfort and mechanical performance of recycled fiber knitted fabrics with virgin ones [27,28,29]. While the point of discussion remains the same, the potential of such knitted fabrics towards functional applications, i.e., antibacterial textiles has not been explored. The previous literature also lacks studies identifying the influence of varying blend ratios of recycled and virgin cotton/polyester fibers on antibacterial properties. Hence, this research focuses on the development of single jersey knitted fabrics using recycled and virgin yarns with blends of cotton and polyester.

Metal oxide nanoparticles are materials of growing interest for drug delivery. Many types of nanoparticles, e.g., zinc oxide (ZnO), magnesium oxides, and titanium dioxides (TiO_2_), etc., have shown antibacterial properties against a broad spectrum of microorganisms. However, there have been no studies on the efficacy of the combined use of recycled textiles and metal oxide nanoparticles to provide antibacterial properties. Hence, in this study, copper, magnesium, and silver finishes were applied through dip coating to incorporate antibacterial attributes into recycled yarns and knitted fabrics. Thermo-physiological comfort and mechanical characteristics were also assessed to check the suitability of the designed antibacterial knitwear in maintaining clothing–body heat equilibrium and service life factors. This study consists of a two-fold approach. On one hand, the effect of the blending percentage of virgin and recycled fibers was studied; on the other hand, the effect of an antibacterial finish on the functional properties was investigated. In addition to this, the fabric was treated with antibacterial finish to check the effects of finishes on the mechanical and thermo-physiological comfort properties of fabric samples.

## 2. Materials and Methods

### 2.1. Materials

**Figure 1:**
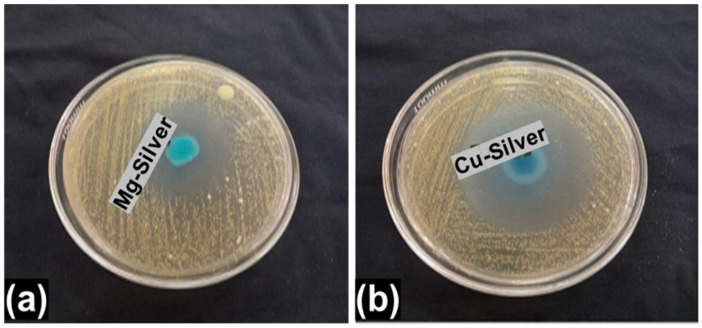
Antibacterial performance of employed finishes: (**a**) magnesium–silver; (**b**) copper–silver.

Cotton and polyester are among the most recycled fibers through mechanical recycling methods. Mechanically recycled cotton and polyester yarns were manufactured with different blend ratios as shown in Table 1. Recycled cotton and cotton/polyester yarns were sourced from Combined Fabrics Limited (Lahore, Pakistan). The recycled polyester was purchased from a local yarn market in Faisalabad, Pakistan. The study also aimed to analyze the influence of antibacterial finishes on the physical attributes of yarns. Hence, the physical characteristics of employed yarns have been described in Table 2. The results presented are averaged from 25 measurements with standard deviation. Two different antibacterial finishes, i.e., copper and silver (CS) nano-finish (50:50) and magnesium and silver (MS) nano-finish (50:50), with 1% and 3% concentrations were employed to analyze the antibacterial function. These finishes were supplied by ZAMVIR, Tech. Fab. International (Faisalabad, Pakistan). Figure 1 shows the antibacterial performances of the employed finishes.

### 2.2. Fabrication Method and Experimental Plan

**Figure 2:**
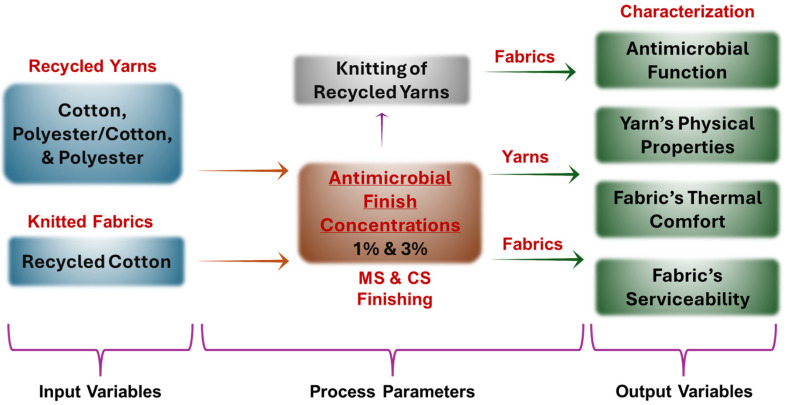
Overall research methodology.

The study consisted of a two-step experimental plan. In the first step, yarns were treated with finishes to analyze the antibacterial performance and the impact of the finishes on physical attributes of yarns. In the second step, these yarns were converted into knitted fabrics, and their antibacterial, thermo-physiological comfort, and mechanical properties were analyzed. In addition to these, knitted fabrics were engineered using recycled cotton yarn, and the finish was applied onto the fabric instead of the yarn to analyze the performance difference between coated yarn and coated fabric specimens. Figure 2 highlights the overall research methodology. A detailed experimental plan is also shown in Table 3. Yarns were converted into knitted fabrics using single jersey structures consisting of all knit stitches with plain architecture on a sample knitting machine (Karlmeyer, Obertshausen, Germany). The machine supports a yarn range of 18 Ne to 36 Ne. Hence, all three yarns smoothly functioned during the knitting process.

#### Pretreatments and Antibacterial Finishing Process

All recycled yarns were processed in the form of yarn lea through a wet pretreatment (solomatic bleaching) process. A textile bleaching process that combines scouring and bleaching into a single step was used to remove any inherent impurities and colorants from the fiber surfaces. Scouring and bleaching were carried out through a batchwise/exhaust method using specific recipes of hydrogen peroxide (H_2_O_2_) and sodium hydroxide (NaOH)/sodium carbonate (Na_2_CO_3_) with a wetting agent and a stabilizer with a material-to-liquor ratio 1:15, at a temperature of 95–100 °C for 30 min, followed by hot and cold rinsing, as well as neutralization using 1 g/L acetic acid. All of the reagents were procured from Havel Composites (Prague, Czech Republic). Different concentrations of bleaching agent and alkali/base were kept for each yarn type owing to unique blend ratios. For recycled cotton yarn, 7 g/L H_2_O_2_, 3 g/L NaOH, 1 g/L wetting agent, and 2 g/L stabilizer were used. For recycled polyester/cotton yarn, 5 g/L H_2_O_2_, 2 g/L NaOH, 1 g/L wetting agent, and 1.5 g/L stabilizer were used. For recycled polyester yarn, 2 g/L Na_2_CO_3_, 1 g/L wetting agent, and 2 g/L detergent were used. These concentrations were based on supplier specifications. The wet pretreatments cleaned the fiber surfaces, facilitating more penetration and adhesion for the antibacterial finish. The antibacterial nano-finishes were also applied on pretreated leas of yarn and knitted fabric samples through a batchwise/exhaust method. Aqueous solutions of the antibacterial nano-finishes were prepared in distilled water for two different concentrations: 1% and 3% *w*/*v*, respectively. Pres and knitted fabric samples were immersed in the prepared solutions and heated to raise the temperature. The process was continued for 30 min at 80 °C for uniform application and fixation of the finish followed by hydro-extracting without rinsing and drying at 100 °C.

### 2.3. Characterization

#### 2.3.1. Physical and Mechanical Properties of Yarn

The physical properties of yarns provide insights into various attributes determining behaviors during fabric manufacturing and in-service life. Twist per Inch/2.5 cm (TPI) describes the amount of twist per unit length and was measured using a digital TPI tester and the ASTM D1422 standard untwist/retwist test method [30]. TPI plays an important role in the softness and crispiness of yarn; hence, it was crucial to analyze. The count lea (120 yards/109.73 m of yarn) strength product (CLSP) is an important mechanical attribute of yarn that predicts the tensile properties of yarn when it is converted into fabric form in the future. CLSP was evaluated on a lea strength tester using the ASTM D1578 standard test method [31]. However, for CLSP evaluation, the yarn needed to be converted into lea form; hence, a warping reel was employed for lea formation by following the procedure described under the ASTM D1578 standard method. Single yarn strength, on the other hand, is also an important mechanical attribute of yarn, providing detailed comprehension of breaking force and elongation. Hence, single yarn strength was determined using an Uster Tensorapid (Uster, Switzerland) following the ASTM D2256 standard test method [32]. All measurements were performed 25 times per each sample. The results were found to be statistically significant with a coefficient of variation lower than 5%.

#### 2.3.2. Fourier Transform Infrared Spectroscopy

The surface modification of knitted fabric samples was assessed by their Fourier transform infrared (FTIR) spectra. The FTIR spectra of fabric samples were recorded on an attenuated total reflectance–Fourier transform infrared (ATR-FTIR) spectrometer (spectrum TWO, PerkinElmer, Waltham, MA, USA), at room temperature in the spectra range between 4000 and 600 cm^−1^, using the ATR reflection technique on an adapter with a crystal of ZnSe. The spectra were collected as a result of 32 running scans at a resolution of 1 cm^−1^.

#### 2.3.3. Scanning Electron Microscopy (SEM)

The surface morphology of knitted fabric samples was examined by scanning electron microscopy (Thermo Scientific Phenom Pure G6 Desktop SEM (GH Eindhoven, The Netherlands). Before SEM analysis, the surface of the fabric sample was sputter-coated with gold (a conductive layer). The images were taken at different suitable accelerating voltages and magnifications with a slow scanning speed to obtain higher-quality images.

#### 2.3.4. Antibacterial Efficacy

The antibacterial efficacy of the applied nano-finishes on the knitted fabric samples against a fresh bacterial strain of *Staphylococcus aureus* (*S. aureus*) was evaluated qualitatively using the agar diffusion method according to the test method AATCC 147 [33]. The swatches of treated fabrics with known dimensions were sterilized (in an autoclave at 121 °C for 15 min) and placed in the center of agar plates and inoculated with the test bacteria. Then, the plates were incubated in a thermostat environment at 37 ± 2 °C for 24 h. After the incubation period, the diameter of the growth-free zone (zone of inhibition) around the fabric and the bacterial growth under/around the fabric were identified.

#### 2.3.5. Washing Durability

The finished knitted fabrics were laundered/washed in an Electrolux-wascator (a front-loading, horizontal rotating drum-type standard reference washing machine, (Stockholm, Sweden), according to the standard test method ISO 6330 [34]. The standard reference detergent (20 g) and a washing temperature of 40 °C were used as per ISO 6330. The washing was conducted for up to 15 wash/laundry cycles, each of which comprised standard washing and rinsing steps. In order to inspect the durability of the antibacterial nano-finish films/coatings to washing, all of the washed fabric samples were subsequently evaluated for antibacterial performance after 1, 5, 10, and 15 cycles following the standard test method AATCC 147.

#### 2.3.6. Thermo-Physiological Comfort and Serviceability of Knitted Fabrics

Thermo-physiological comfort attributes are vital to ensure the prolonged wearing of textile materials. Knitted fabrics unable to maintain thermal equilibrium between wearer and clothing are also not suitable for functional applications. Hence, thermo-physiological comfort properties including air permeability and moisture management were characterized.

Air permeability is the property of a fabric to allow air to pass through it. Fabrics used in bodywear require reasonably good air permeability. It is directly linked to breathability and comfort. On the other hand, low air permeability is also required in particular fields, including protective and safety clothing. Air permeability was measured under the ISO 9237 international standard test method [35]. The commercial air permeability tester FX 3300 Lab Air IV testing instrument (Textest AG, Schwerzenbach, Switzerland) was used, working according to ISO 9237. A laminar flow of air was calculated in mm/s through a specific area of the fabric at a pressure of 100 Pa. The area of measurement was 100 cm^2^ and the measurements were repeated 10 times for each sample to calculate the average. The results were found to be statistically significant with a coefficient of variation lower than 5%.

Overall moisture management capability was characterized in terms of the OMMC index by following the AATCC 195 standard test method [36]. The OMMC index provides insights into overall moisture handling performance in a single value instead of individual parameters, i.e., water vapor permeability, wetting time, wetting speed, etc. OMMC analyzes the management and transport of liquid moisture through the surface and the bulk of the fabric. It was tested on a moisture management tester, MMT (SDL ATLAS, Rock Hill, SC, USA). The samples tested using the MMT device have a standardized size of 80 mm × 80 mm. Each measurement lasted 120 s. During the first 20 s, a water drop was applied to the upper surface of the sample. The water drop was applied onto the center of the upper side of the sample. The applied drop was transported through the sample in 3 directions: on the upper surface of the fabric; through the fabric from the upper surface to the lower surface; on the lower surface of the fabric. Considering these factors, the various moisture management parameters listed below were evaluated.

1. Wetting time of top surface (s) denoted as WTT;
2. Wetting time of bottom surface (s) denoted as WTB;
3. Absorption rate of top surface (%/s) denoted as TAR;
4. Absorption rate of bottom surface (%/s) denoted as BAR;
5. Maximum wetted radius for top surface (mm) denoted as MWRT;
6. Maximum wetted radius for bottom surface (mm) denoted as MWRB;
7. Spreading speed on top surface (mm/s) denoted as SST;
8. Spreading speed on bottom surface (mm/s) denoted as SSB;
9. Accumulative one-way transport index (−) denoted as R;
10. Overall moisture management capability (−) denoted as OMMC.

All of the tests were performed under standard testing conditions, e.g., a temperature of 21 ± 2 °C and a relative humidity of 65 ± 2%. Ten measurements were taken for each sample, and the average was calculated. The results were found to be statistically significant with a coefficient of variation lower than 5%.

Bursting strength ensures the serviceability of knitted fabrics, as the fabrics encounter several multiaxial stresses caused by the wearer’s body and the environment during their service life. It refers to the force required to break or rupture the fabric under hydraulic or pneumatic pressure. To evaluate the bursting strength of the fabrics, the sample size used was 112 mm^2^ using the standard method of ASTM D3786 [37]. The samples were clamped over a rubber diaphragm, and a hydraulic bursting strength tester was used to calculate the bursting strength. During the test, the samples were clamped over the rubber diaphragm and fluid pressure was applied to the samples until bursting. The value of bursting pressure for each sample was measured 10 times, and the average was calculated.

## 3. Results and Discussion

### 3.1. Physical and Mechanical Characteristics of Yarns

**Figure 3:**
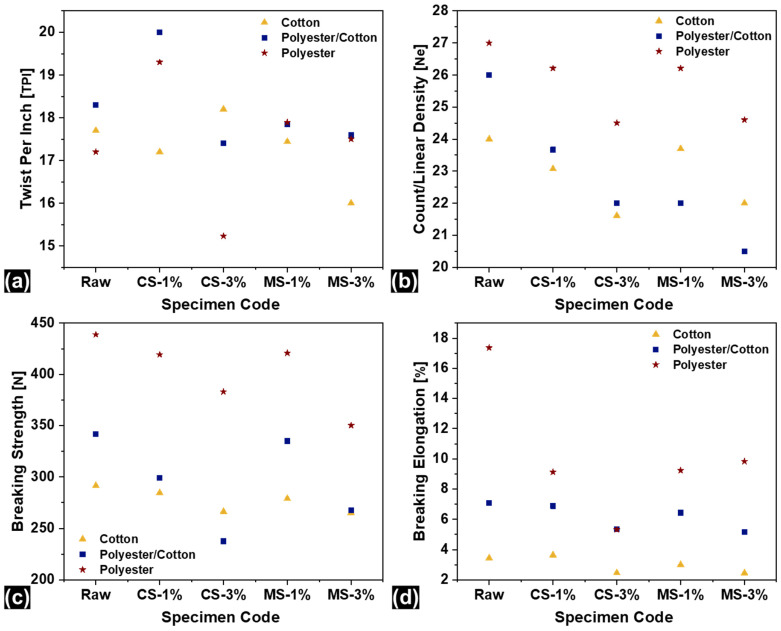
Physical and mechanical properties of recycled yarns, (**a**) twist per inch (2.5 cm); (**b**) count/linear density; (**c**) breaking strength; (**d**) breaking elongation.

The specialty finishing process influences the physical and mechanical attributes of yarns. Fibers can swell, the surface can become smooth and slippery, and inter-fiber frictions can vary owing to finish absorption [38]. Hence, it was crucial to analyze the above-mentioned properties. Figure 3a shows the results of TPI for raw and finished yarn specimens.

Samples of 100% cotton yarn exhibited a different trend from polyester/cotton and 100% polyester yarns. Polyester/cotton and 100% polyester showed increases of about 9.28 ± 0.21% and 12.20 ± 0.33% in TPI, respectively, at a 1% CS finish concentration, while a decrease of 2.82 ± 0.04% was noted for cotton yarn. Cotton, being a natural fiber, has more affinity to fluids as compared to polyester. Hence, the swelling of cotton fibers caused axial expansion of fibers in the yarn cross-section and the amount of TPI was decreased [39]. Polyester, on the other hand, has a different behavior. The finish made the surface of polyester more slippery, and fibers came closer owing to inter-fiber slippage which made the number of TPIs higher [40]. Moreover, 100% recycled polyester had a higher increase in TPI than polyester/cotton blended specimens. However, a further increase in finish concentration resulted in the opposite behavior. The 100% cotton specimen showed about a 2.83 ± 0.05% increase in the number of TPIs. This is because of the inability of the fibers to absorb more fluid and the slippage of fibers due to wet surfaces which led to an increase in TPI. At 3% CS concentration, polyester started absorbing the finish more effectively, and the fibers’ swelling caused a 4.91 ± 0.12% and 11.23 ± 0.47% decrease in TPI for polyester/cotton and pure polyester, respectively. The polyester/cotton and 100% polyester yarns exhibited a similar trend for MS finish, but the difference in TPI was not much higher than it was for CS-finished specimens. On the other hand, cotton yarn exhibited a different trend for CS finish. A linear decrease in the number of TPIs was noted: a 1.46 ± 0.02% decrease in TPI was noted at 1% MS concentration, and 3% MS concentration resulted in a further 9.60 ± 0.35% decrease. In fact, all of the yarns were prepared with similar twist levels in order to make them comparable in terms of mechanical and transmission-related performance. Therefore, the results of TPI seem similar. However, the increase in TPI after finishing is slightly variable depending on the yarn type and percentage of finishing. The results of TPI were found to be statistically significant with a coefficient of variation lower than 5%.

Linear density characterization also revealed almost similar trends for cotton and polyester yarns as shown in Figure 3b. The increase and decrease in linear densities is attributed to swollen fibers after deposition of the nanoparticles and a change in the linear density (an indirect yarn count system has been used in this research). Therefore, the yarns became coarser [41]. The same phenomenon was observed in characterized specimens where fibers swelled owing to finish absorption making the yarn coarser. An interesting fact was noted that increasing concentrations of both CS and MS finishes caused an increase in yarn coarseness. Hence, it was assumed that more absorption of the finish occurred in yarns at elevated concentrations. For example, cotton yarn showed a 6.32 ± 0.24% lower linear density for CS-3% as compared to CS-1%. The trends of cotton and polyester specimens were similar. However, polyester/cotton yarn had a different behavior. A linear decrease in yarn fineness/count can be observed from raw specimens towards MS-3% in Figure 3b. This fact confirms the higher affinity of polyester/cotton blended yarn to MS finish which caused about a decrease of 21.15 ± 1.02% in linear density with MS-3% as compared to the raw specimen. The results were found to be statistically significant with a coefficient of variation lower than 5%.

The mechanical properties of yarns also seemed to be affected by the antibacterial finishing process. Figure 3c,d highlight the breaking strength and elongation of mechanically characterized specimens after the single yarn strength test. Most chemical finishing treatments (except softener finishing) cause yarns to be stiffer, and breaking strength and elongation are usually compromised. The applied antibacterial finish worked in a similar manner, causing the breaking strength and elongation of finished specimens to be lower than those of untreated/bleached-only samples. Cotton, being a natural fiber, had different interactions with the antibacterial finishes as compared to polyester. Hence, a smaller change in strength and elongation were observed for cotton yarns before and after finishing. The CS-1% specimen exhibited 2.40 ± 0.02% lower strength than untreated/bleached-only cotton samples, and the increased concentration of CS-3% caused a further 8.64 ± 0.22% decrease in yarn strength. Similar behavior was noted for elongation as well: a maximum difference of 28.48 ± 0.72% was obtained between raw cotton and the CS-3% specimen (Figure 3d). Settlement of unabsorbed antibacterial finish between fibers restricted their movement, making them stiffer, less elastic, and weaker [42]. Polyester, being less hydrophilic than cotton, has more finish between fibers, which is why higher differences in strength and elongation were obtained for polyester and polyester/cotton yarns. Polyester/cotton yarn showed a 12.43 ± 0.23% decrease in strength for CS-1%, which was only 2.40 ± 0.03% for pure cotton. The increasing concentration caused a 30.49 ± 1.08% decrease in strength. Similar behaviors of cotton and polyester were noted for MS-finished specimens, i.e., MS-3% finished polyester offered 20.20 ± 1.04% lower strength than untreated/bleached-only samples of recycled 100% polyester. The facts proved that the mechanical properties of polyester fiber were more adversely affected by antibacterial finishing possibly due to more finish accumulation and curing between fibers. Moreover, the elongation of polyester showed an exponential decrease after finishing, which can be observed in Figure 3d. The results were found to be statistically significant with a coefficient of variation lower than 5%.

### 3.2. Weight Gain of Knitted Fabrics

In textile chemical finishing processes, there are different desired end uses; the aesthetic and functional properties presented by the textiles depend on the amount of chemical/finish deposited on the textile substrates. Therefore, the weight gain of the knitted fabrics was assessed after treatment with aqueous antibacterial nano-finish solutions of different concentrations. The effect of different concentrations of finishing solutions on the weight gain of knitted fabrics is given in Table 4. The weights (gram per square meter) of untreated/bleached-only samples of cotton, polyester–cotton, and polyester were almost similar ~140 g/m^2^. The weight was found to increase, and this effect was observed more strongly when the finish was applied on knitted fabric samples. However, the weight gain was also found to increase with an increase in finish concentration due to the maximum wetting and binding of individual fibers and yarns and ultimately retaining of the finish (nanoparticles) in the fabric structure after hydro-extracting and drying. All measurements were repeated 10 times and the CV% was lower than 5%.

### 3.3. Fourier Transform Infrared Spectroscopy

**Figure 4:**
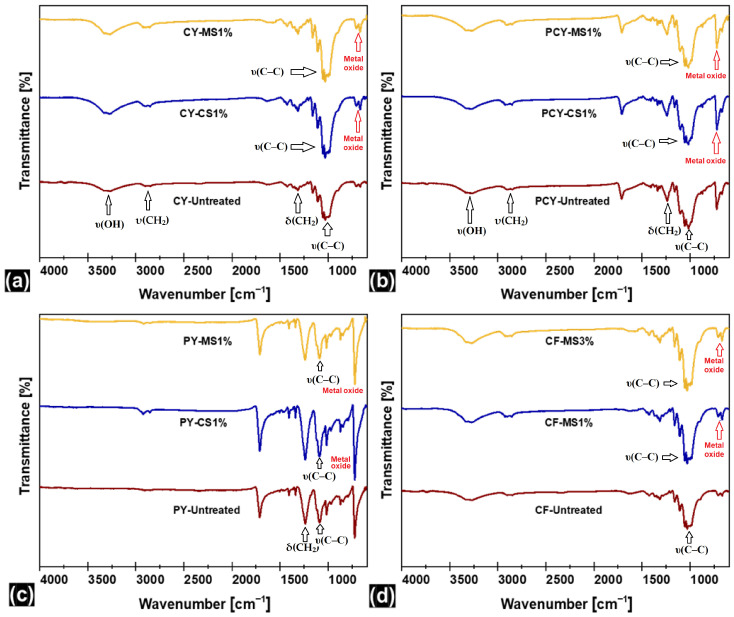
FTIR spectrums of antibacterial finished specimens: (**a**) CY specimens; (**b**) PCY specimens; (**c**) PY specimens; (**d**) CF specimens.

The modification of the knitted fabric surface was investigated by ATR-FTIR spectroscopy to find the molecules present in the samples and their functional groups. The ATR-FTIR spectra of untreated/bleached-only samples and finished knitted fabrics are shown in Figure 4, which affirmed the presence of numerous absorption peaks as an indication of different functional groups.

FTIR spectra of finished yarn and fabric samples were critically analyzed and verified with the literature [43,44,45,46,47] to extract valuable information about the nature of the nanoparticles present in the finish formulations. The antibacterial finishes were produced using nanocluster technology as claimed by the manufacturer/supplier. The present innovative product relates to the emulsion of nanoparticles in stable aqueous dispersions of metal/metal oxides nanoclusters containing many other chemical auxiliaries using green nanochemistry.

In the case of the untreated/bleached-only sample fabrics of cotton and polyester–cotton, the characteristic vibration modes of cellulose were detected, i.e., υ(OH) at ca. ~3300, υ(CH_2_) at ~2900, δ(OH) at ~1640, δ(CH_2_) at ~1425, δ(CH) at ~1370, δ(OH) at ~1310, υ(C–C) at ~1020, and υ(OH) at ~894 cm^−1^, and the characteristic vibration modes of polyester were detected, i.e., υ(C=O) at 1720 cm^−1^, υ(C-H) at 2920 cm^−1^ and 2880 cm^−1^, and υ(C-O) at 1250 cm^−1^ [48]. Metal nanoparticles have also their own characteristic vibration peaks. Apart from these, the prominent peaks of lattice vibrations of M−O, M−O−M, and O−M−O (M stands for metal) were observed in the low-energy region of the spectra (~500–1000 cm^−1^), manifesting the presence of copper, magnesium, and silver metal nanoparticles [49,50,51]. The FTIR spectra profiles of finished fabrics are very similar to those of untreated/bleached-only samples. However, an increase in the size/intensity of peaks is a direct indication of the amount of antibacterial nano-finish material present.

### 3.4. Scanning Electron Microscopy

**Figure 5:**
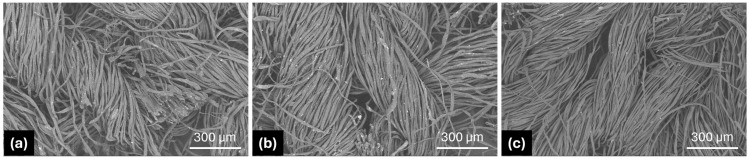
SEM images of specimens: (**a**) CF-untreated/bleached-only sample; (**b**) CY-MS-3%; (**c**) CF-MS3%.

The overall morphology of the knitted fabric surface appearance was inspected by scanning electron microscopy (SEM). Typical SEM images of the untreated/bleached-only samples and finished knitted fabrics are shown in Figure 5, displaying the presence of antibacterial nano-finishes on fabric samples for effective antibacterial activity. The homogeneous distribution of antibacterial nano-finishes on the knitted fabric samples uniformly deposited and modified the surface of the textile substrates (i.e., fibers, yarns, and fabrics) without any accumulation of finish as evidenced from the SEM images. Figure 5a shows the untreated/bleached-only sample without any nano-finish. Figure 5b,c depict SEM images of knitted fabric samples, i.e., finished after knitting and prepared from finished yarn, respectively, which were compared to examine how the finish system was laid on the textile substrate. It can be clearly observed from Figure 5b that the finish is introduced into the yarn as a thin film/coating covering the fibers/yarns but not the surface of the fabric. Contrarily, the fabric that was finished after knitting exhibited a thin film/coating covering the fibers/yarns as well as the surface of the fabric, as evidenced from Figure 5c. Uniform deposition of the finish was found due to the maximum wetting and binding of individual fibers/yarns that also resulted in partial covering or filling of the inter-fiber and inter-yarn spaces/pores of loops/stitches by the finishing of the fabric after knitting. This leads to deterioration in the wearing comfort of fabric samples finished after knitting.

### 3.5. Antibacterial Efficacy

**Figure 6:**
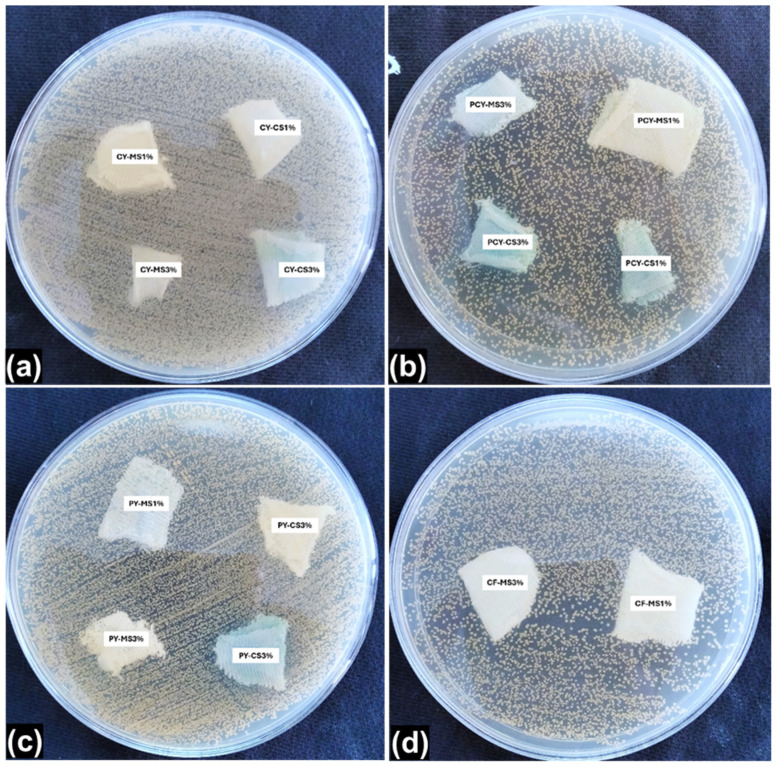
Agar plate images during antibacterial activity: (**a**) CY specimens; (**b**) PCY specimens; (**c**) PY specimens; (**d**) CF specimens.

**Figure 7:**
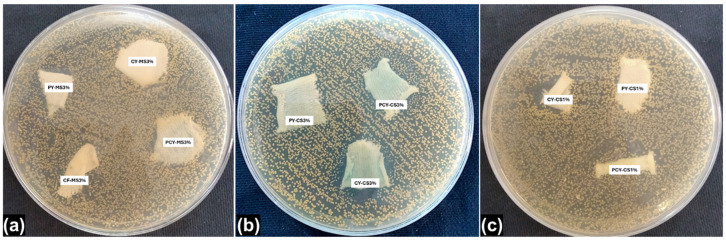
Agar plate images during antibacterial activity: (**a**) MS-3% specimens; (**b**) CS-3% specimens; (**c**) CS-1% specimens.

The antibacterial nano-finishes were purchased from a company. Therefore, they were first evaluated to ensure their antibacterial activity, and the inhibition zone was clearly observed which confirmed their efficacy (also already presented in Figure 1). Then, on the basis of these findings further work was conducted. The formation of the inhibition zone is the most widely accepted visual fact relating to the antibacterial activity of any finished textile substrates. But sometimes a clear inhibition zone does not appear around the finished fabric samples due to non-/poor leaching properties of the antibacterial finishes. In that situation, the other most important truth/criterion for evaluation of antibacterial activity is the presence/absence of bacterial colonies growing under or around the finished fabric samples. In this study, antibacterial activity was present and observed on the basis of the killing of bacterial strains by the finished fabric samples before and after washing, i.e., the presence/absence of bacterial colonies. The overall antibacterial efficacy results are given in Table 5 and Table 6 and Figure 6 and Figure 7.

The in vitro antibacterial activity of knitted fabrics was determined by testing against the *Staphylococcus aureus* (*S. aureus*) bacterial strain. After the incubation period, the inhibition zone and bacterial growth were assessed for each fabric sample to compare the antibacterial efficacy. The untreated/bleached-only fabric samples revealed no antibacterial activity against *S. aureus*; the presence of colonies can be seen on the surface, thus indicating bacterial growth. The visual aspects of the antibacterial tests are shown in Figure 6.

The results revealed that most of the finished fabric samples showed an antibacterial effect. The finished fabric samples placed on the bacteria-inoculated surfaces killed all of the bacteria under and around the region of the fabric. It also demonstrated the distinct inhibition of bacterial growth around finished fabric samples for *S. aureus*. The copper–silver-based antibacterial nano-finish exhibited a higher inhibitory activity against *S. aureus* as compared to that of the magnesium–silver-based antibacterial nano-finish. However, a higher inhibition of bacterial growth was obtained for a higher concentration of each antibacterial nano-finish, emphasizing the influence of concentration.

Antibacterial finishes and different metal nanoparticles (such as copper, magnesium, silver, titanium, zinc, etc.) have been validated as potent antibacterial agents, due to their effectiveness in microbial resistance through different proposed mechanisms to oppose microbial growth. They inhibit growth or preferably kill microorganisms by several different mechanisms which act around the cell wall of microorganisms. Thus, cell wall damage, alteration of cytoplasm membrane permeability, alteration of physical or chemical state of nucleic acids and proteins, inhibition of enzyme action, or inhibition of nucleic acid or protein synthesis are all physical and chemical approaches that can be employed by antibacterial finishes to inhibit growth or kill microorganisms [52,53,54].

The most important properties of textile treatments are durability and washing fastness. Washing procedures usually cause a reduction in antibacterial activity. In general, the effectiveness of the finished material decreases and, in parallel, a reduction in bacterial viability can be observed as shown in Figure 7.

In this study, antibacterial activity was present and observed on the basis of the killing of bacterial strains by the finished fabric samples before and after washing, i.e., the presence/absence of bacterial colonies’ growth. Since the antibacterial activity was qualitatively evaluated, only the presence or absence of the bacterial colony was visualized. The results of wash durability after 1, 5, 10, and 15 cycles are given in Table 6.

After 15 washing cycles, there was a change in the bactericidal activity of the knitted fabric samples finished with 1% antibacterial nano-finish concentrations, as summarized in Table 6.

### 3.6. Air Permeability of Knitted Fabrics

**Figure 8:**
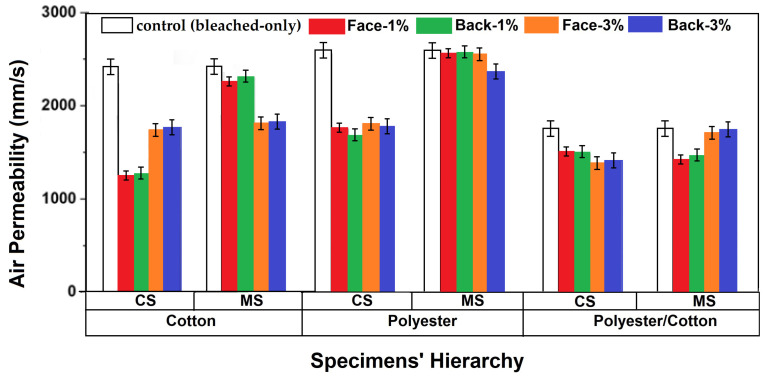
Air permeability of knitted fabrics.

Maintaining adequate air permeability is necessary for antibacterial knitted fabrics along with their functional attributes. Usually, antibacterial knitwear is worn for prolonged times in order to perform its core functions. Hence, if air permeability is not maintained, the thermal equilibrium of the body is disturbed, and a sense of discomfort develops. This is why it was crucial to characterize the developed specimens in terms of air permeability, the results of which are detailed in Figure 8. It is well established that air permeability decreases after the wet finishing of fabrics [55,56,57,58]. This is attributed to swelling of the fibers and yarns which causes a shrinkage in the knitted fabric. Therefore, the overall porosity decreases, reducing the ability to allow air passage [51,52].

**Figure 9:**
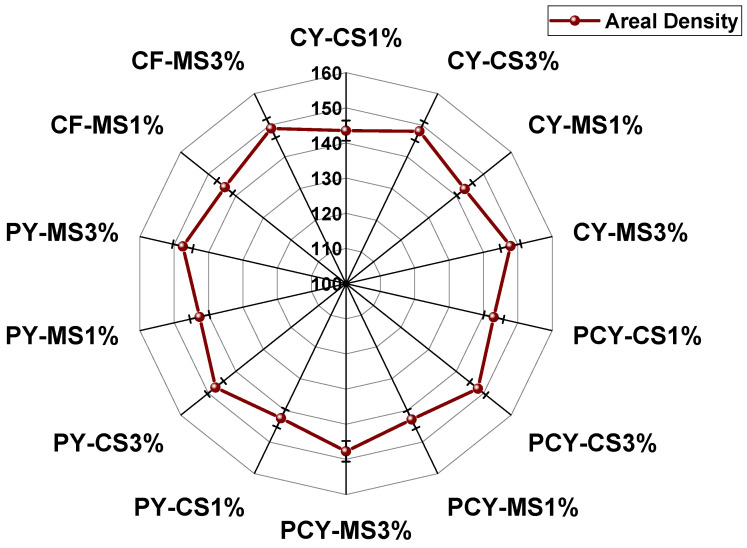
Areal density of knitted fabrics.

Air permeabilities were separately characterized on the face and back surfaces for the finished samples. However, the obtained values were almost equivalent for all specimens. Changing the finish concentration developed more prominent air permeability variation for cotton knitted fabrics. Increasing the concentration of CS finish increased air permeability by about 41.02 ± 2.01% instead of the increasing areal density/grams per square meter of fabric (Figure 9).

However, the trend was found to be different for MS-finished fabrics, where increasing MS concentration to 3% resulted in a 19.77 ± 0.78% decrease in the air permeability of cotton knitted fabrics. The phenomenon was governed by an increasing number of TPIs at CS-3% (Figure 3a) which made the yarns tighter and crispier. Thus, the inter-yarn pores through the loops in the knitted structure became more open and allowed more air to pass through the fabric. The number of TPIs decreased for MS-3% specimens (Figure 3a); hence, air permeability decreased with an increasing concentration of MS finish. However, polyester knitted specimens also exhibited a similar trend to the cotton fiber-based sample. The 3% CS-finished specimen showed a 5.80 ± 0.12% higher air permeability than the 1% CS-finished specimen. The 3% MS-finished fabric also had a 7.92 ± 0.15% lower air permeability as compared to the 1% concentration. Hence, it was evident that the inherent behaviors of antibacterial finishes also affect air permeabilities. However, polyester/cotton blended fabrics exhibited totally opposite behavior. Increasing CS concentration decreased air permeability, and vice versa for MS-finished knitted fabrics. The blending of fibers not only showed a significant influence on changing trends, but also decreased the overall air permeability values. The highest air permeability of the polyester/cotton 3% MS-finished specimen was 26.72 ± 0.91% lower than the 3% MS-finished specimen of polyester fiber. Another factor under consideration was to compare the air permeability of yarn-finished antibacterial fabrics with specimens finished at the fabric stage. Curing of the antibacterial finish occurred between inter- and intra-loop spaces when cotton fabric was being treated. The phenomenon decreased the porosity of the fabric. Hence, the air permeabilities of specimens treated at the fabric stage were found to be much lower than samples from treated yarn. The fabric sample treated with CS-1% exhibited a 47.76 ± 1.58% lower air permeability than CS-1% yarn-finished fabric sample. Such a phenomenon proved that antibacterial treatment at the yarn stage was more effective in maintaining higher air permeability.

**Figure 10:**
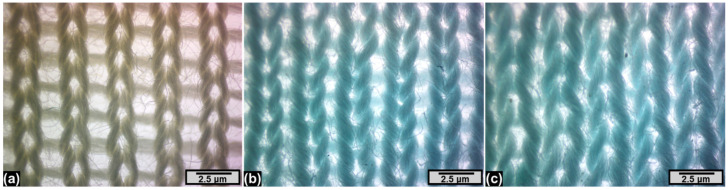
Photographic analysis of specimens: (**a**) unfinished raw fabric; (**b**) yarn-finished fabric; (**c**) finish applied after knitting.

Figure 10 shows visual images of untreated/bleached-only fabric samples (Figure 10a), samples treated at the yarn stage (Figure 10b), and samples treated at the fabric stage (Figure 10c).

It is visible that the porosity of the untreated/bleached-only sample is the maximum, followed by the sample made from finished yarns and the sample finished at the fabric stage. The swelling of the fibers and yarns can also be observed in Figure 10b,c.

### 3.7. OMMC of Knitted Fabrics

OMMC is an indexed result of several moisture management parameters, e.g., water vapor permeability, wetting rate, wetting time, etc. Hence, a single value of the OMMC index encompasses all auxiliary moisture management attributes. Antibacterial textile materials are mostly prone to bodily fluids like sweat and wound exudates. Hence, the fabrics should be capable of managing these fluids and transporting them into external environments. The results of all of the moisture management parameters for the untreated/bleached-only and finished samples are given in Table 7.

**Figure 11:**
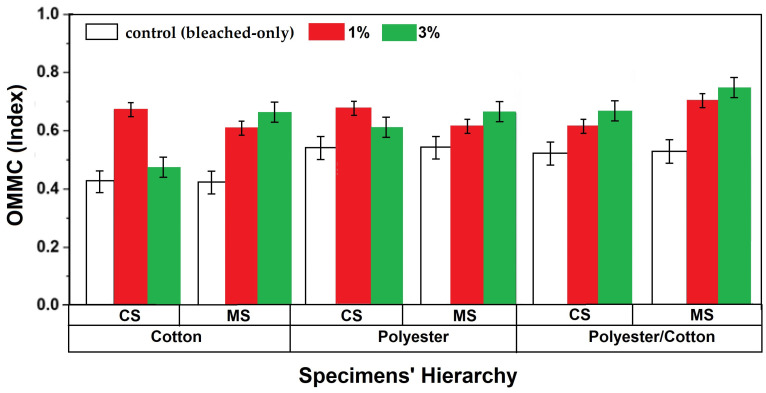
OMMC indices of knitted fabrics.

The OMMC index values of the developed specimens were compared with the untreated/bleached-only sample. The results are shown in Figure 11.

In general, the OMMC index depends on two major factors: firstly, the hydrophilicity of the material itself to absorb/adsorb the moisture; secondly, the fabric porosities supporting the moisture management. The knitted structures and other knitting parameters were kept constant in this research. Hence, the trends observed in the OMMC results were governed by the fiber type, yarn type, and finish applied. It was observed that the OMMC values for the treated samples were higher than those of the untreated/bleached-only samples. This can be attributed to the micro channels created by the finishing treatment which allow capillary action and transportation of water molecules through the inter-fiber channels. Such observations have also been reported by several researchers [48,55]. In several cases, the finishing treatment with higher concentration also facilitated better moisture management due to higher adsorption of water molecules on the nanoparticles deposited on the fiber/fabric surface [56]. The 100% cotton and 100% polyester knitted fabrics exhibited a decrease in OMMC index with an increasing concentration of CS finish. The cotton specimen showed a 30.88 ± 1.12% decrease, while polyester fabric showed a 10.29 ± 0.25% lower OMMC index for CS-3% as compared to fabrics treated with a 1% concentration of the finish. Increasing the concentration of antibacterial finish decreased the inherent fabric porosities, and CS finish could not handle moisture appropriately which resulted in a compromised OMMC index. The MS-finish-treated specimens showed a different trend of an increasing OMMC index with increasing finish concentration. The MS-3% treated cotton yarn specimen has a 8.19 ± 0.17% higher OMMC index than the 1% concentration of MS, and the same difference was noticed for 100% polyester knitted fabrics. Cotton knitted fabric was also treated at the fabric stage with MS finish for comparison with yarn-finished knitted fabric. The results also proved the affinity of MS finish towards moisture. The fabric-stage-treated cotton fabric exhibited the highest OMMC index of 0.8 among all other specimens; the value was 21.21 ± 0.88% higher than yarn-finished specimens of cotton. Polyester/cotton blended yarn knitted fabrics on the other hand showed a linear increase in OMMC indices with increasing concentrations for both finish types. The inherent moisture handling behavior of the fiber and finishing material remains the dominating parameter up to a 3% concentration of CS finish, leading to an elevated moisture management index. A similar phenomenon was seen for MS-finished fabrics and increased the OMMC index by 1.35 ± 0.04%. The polyester/cotton blended yarn knitted specimens offered overall higher moisture management as compared to other yarn-finished specimens, proving blending to be an efficient solution to impart better moisture management attributes irrespective of the finish type or concentration employed.

### 3.8. Bursting Strength

**Figure 12:**
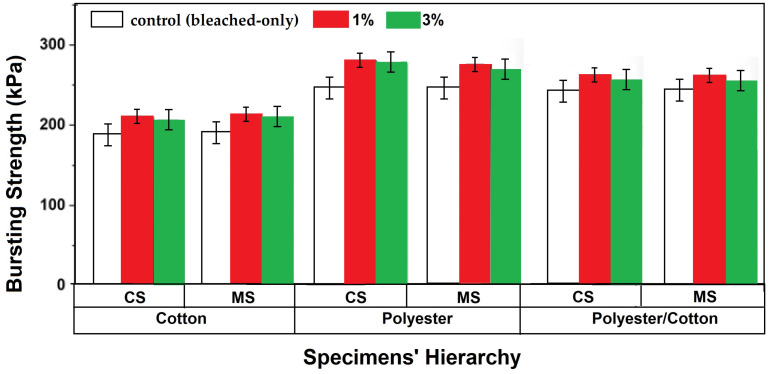
Bursting strength of knitted fabrics.

Bursting strength is a crucial indicator determining the serviceability of knitted fabrics. Knitwear fabrics undergo several multidirectional environmental stresses which can cause ruptures or bursts in the influenced area. Hence, bursting performance was characterized in this research, providing insights into fabric serviceability. Figure 12 highlights the results of the characterized specimens. Bursting strength was measured at each concentration of applied antibacterial finish to analyze the influence of changing the finish type and concentration on serviceability.

The bursting strength values of the finished samples were slightly higher than those of the untreated/bleached-only samples [27,48]. This can be attributed to the cross-linking of the nanoparticles on the fiber surfaces, which improves the inter-fiber friction and thus the resistance to bursting of the yarns and fabric [45,57]. On the other hand, a linear trend of a slight decrease in bursting strength was noticed for all specimens with an increase in antibacterial finish concentration from 1% to 3%. This could be due to the overload of nanoparticles which deteriorate the bursting resistance of the yarns and fabric [44,56], though the overall bursting resistance/strength in all finished samples was still higher than that of the untreated/bleached-only sample. The influence of fiber-inherent mechanical properties remained dominant, i.e., polyester was stronger as a synthetic fiber which resulted in the highest bursting strength followed by a decreasing trend towards the polyester/cotton blend and cotton. The highest bursting strength of 280 kPa was observed for recycled polyester fabrics having a 1% concentration of CS finish. However, MS-finished fabrics having a similar concentration resulted in a 1.78 ± 0.04% lower bursting strength. Bursting strength, being dependent on the mechanical attributes of yarns, corresponded to the single yarn strength results shown in Figure 3c. The strength of MS-finished polyester yarns was relatively lower than that of CS-finished ones. Hence, the bursting strength of the corresponding fabrics samples was observed to be lower. The cotton sample showed about a 0.9 ± 0.02% higher bursting strength for MS-finished fabrics, and a similar performance was noticed for polyester/cotton blended yarn knitted fabrics. The contribution of both polyester and cotton in a single yarn balanced the bursting performance. The trend of decreasing bursting strength with increasing concentrations remained similar for all yarn types owing to a lower single yarn strength for 3% finished specimens (Figure 3c). The fabric-finished cotton specimens exhibited a higher bursting strength as compared to the yarn-finished cotton fabrics. This phenomenon can be attributed to the enhanced compactness and links between interloping zones supported by the antibacterial finish. Also, the swelling of yarns visible in Figure 10b,c is responsible for the compactness and increase in bursting resistance. Finish application at the fabric stage improved the bursting performance by 2.8 ± 0.11% and 2.4 ± 0.05% for 1% and 3% finish concentrations, respectively.

## 4. Conclusions

In this research, two unique approaches were successfully combined to develop recycled textiles for value-added medical textile products. The antibacterial applications do not require extreme mechanical attributes. Hence, the recycled yarns were found to be suitable for such applications.

There was an increase in TPI after finishing depending on the yarn type and the percentage of finishing. The swelling of fibers after the deposition of nanoparticles also increased the linear density. The chemical finishing treatments caused the yarns to be stiffer, and breaking strength and elongation were compromised. The applied antibacterial finish worked in a similar manner, causing the breaking strength and elongation of finished specimens to be lower than those of untreated/bleached-only samples. The results were found to be statistically significant with a coefficient of variation lower than 5%.

Using a two-fold approach, the antibacterial finishes were applied at both the yarn and fabric stages, i.e., before and after knitting. Microscopic and SEM analysis revealed that finish application at the fabric stages contributed to a reduction in porosities. This phenomenon did not affect the antibacterial activity. The antibacterial effect was improved by increasing the concentration from 1% to 3%. The antibacterial finish was still effective after 15 laundering/washing cycles.

The thermo-physiological comfort and serviceability factors were influenced by the finish concentration. The air permeability of finished fabrics was lower than that of untreated/bleached-only samples. For 100% cotton fabrics, increasing the concentration of CS finish increased the air permeability by about 41.02 ± 2.01% instead of increasing the areal density/grams per square meter of fabric. For recycled polyester specimens, increasing the concentration of CS finish from 1% to 3% also increased the air permeabilities, and the opposite was seen for MS finish. However, the polyester/cotton blend showed an inverse trend. The specimens finished at the fabric stage showed lower air permeabilities than yarn-finished specimens. The decreased air permeability was attributed to reduced porosities but worked efficiently for OMMC enhancement, as moisture channels/capillaries were enhanced for the transportation of water through the fibrous material. Therefore, treated samples showed a higher OMMC as compared to untreated/bleached-only samples. In some cases, finishing treatments with a higher concentration also facilitated better moisture management due to better adsorption of water molecules on the nanoparticle surface. Bursting strength was improved by 2.8 ± 0.11% and 2.4 ± 0.05% for 1% and 3% finish concentrations, as the finish created linkages between fibers and more force was required to burst the knitted fabrics multiaxially.

The limitations of the study include the lack of a detailed characterization of the nanoparticles deposited onto the textile material. Such information will be necessary to further understand the influence of specific functional nanoparticles on antibacterial performance.

## Figures and Tables

**Table 1:** Composition of recycled yarns.

Sr. No.	Yarn Type	Blend Ratio
1	Virgin Cotton/Recycled Cotton	60:40
2	Recycled Cotton/Recycled Polyester	63:37
3	Recycled Polyester	100

**Table 2:** Physical properties of recycled yarns (average ± standard deviation).

Sr. No.	Physical Property	Yarn Type		
		Recycled Cotton (C)	Recycled Cotton/Polyester (PC)	Recycled Polyester (P)
1	Count (Ne)	24 ± 2	26 ± 2.1	25 ± 2.1
2	Twist Per Inch (2.5 cm) (TPI)	17.7 ± 1.2	18.3 ± 1.2	17.2 ± 1.1
3	Count Lea Strength Product (CLSP)	2592 ± 70	3595 ± 85	2210 ± 62
4	Single Yarn Strength (N)	291.5 ± 11.2	438.8 ± 18.5	341.7 ± 12.1
5	Elongation (%)	3.44 ± 0.04	17.36 ± 0.52	7.08 ± 0.64
6	Tenacity (cN/Tex)	12.83 ± 0.23	20.06 ± 0.31	13.88 ± 0.3
7	Coefficient of Variation (CV%)	8.94 ± 0.35	8.54 ± 0.22	9.66 ± 0.37

**Table 3:** Experimental plan and specimen codes.

Sr. No.	Specimen Description	Finish Concentration	Specimen Code
1.	Recycled Cotton Yarn–Copper and Silver Nano-finish *	1%	CY-CS1%
2.		3%	CY-CS3%
3.	Recycled Cotton Yarn–Magnesium and Silver Nano-finish *	1%	CY-MS1%
4.		3%	CY-MS3%
5.	Recycled Polyester/Cotton Yarn–Copper and Silver Nano-finish *	1%	PCY-CS1%
6.		3%	PCY-CS3%
7.	Recycled Polyester/Cotton Yarn–Magnesium and Silver Nano-finish *	1%	PCY-MS1%
8.		3%	PCY-MS3%
9.	Recycled Polyester Yarn–Copper and Silver Nano-finish *	1%	PY-CS1%
10.		3%	PY-CS3%
11.	Recycled Polyester Yarn–Magnesium and Silver Nano-finish *	1%	PY-MS1%
12.		3%	PY-MS3%
13.	Recycled Cotton Fabric–Magnesium and Silver Nano-finish **	1%	CF-MS1%
14.		3%	CF-MS3%

* Antibacterial finish was applied on yarns and then knitting was performed. ** Antibacterial finish was applied on knitted fabrics.

**Table 4:** Weight gain of knitted fabrics after antibacterial finish application.

Sr. No.	Specimen Code	Finish Concentration (%)	Weight (g/m^2^)
1	CY-CS1%	1	143.5 ± 4.08
2	CY-CS3%	3	148.1 ± 5.45
3	CY-MS1%	1	143.1 ± 3.74
4	CY-MS3%	3	147.9 ± 4.44
5	PCY-CS1%	1	143.0 ± 2.71
6	PCY-CS3%	3	147.9 ± 4.01
7	PCY-MS1%	1	142.9 ± 6.04
8	PCY-MS3%	3	147.7 ± 5.41
9	PY-CS1%	1	142.5 ± 2.33
10	PY-CS3%	3	147.4 ± 4.42
11	PY-MS1%	1	142.6 ± 3.12
12	PY-MS3%	3	147.5 ± 6.41
13	CF-MS1%	1	144.0 ± 4.22
14	CF-MS3%	3	149.0 ± 5.48

**Table 5:** Antibacterial activity of finished knitted fabrics.

Sr. No.	Specimen Code	Bacterial Growth in Sample Contact Area (Yes/No)	Clear Zone of Inhibition (mm)
1.	CY-CS1%	No	0.00
2.	CY-CS3%	No	0.00
3.	CY-MS1%	Yes	0.00
4.	CY-MS3%	No	0.00
5.	PCY-CS1%	No	0.00
6.	PCY-CS3%	No	0.00
7.	PCY-MS1%	Yes	0.00
8.	PCY-MS3%	No	0.00
9.	PY-CS1%	No	0.00
10.	PY-CS3%	No	0.00
11.	PY-MS1%	Yes	0.00
12.	PY-MS3%	No	0.00
13.	CF-MS1%	Yes	0.00
14.	CF-MS3%	No	0.00

**Table 6:** Antibacterial activity of finished knitted fabrics after 1, 5, 10, and 15 washing cycles.

Sr. No.	Specimen Code	Bacterial Growth in Sample Contact Area (Yes/No) After Washing Cycles				Clear Zone of Inhibition (mm) After Washing Cycles			
Washing Cycles		1	5	10	15	1	5	10	15
1	CY-CS1%	No	No	No	Yes	0.00	0.00	0.00	0.00
2	CY-CS3%	No	No	No	No	0.00	0.00	0.00	0.00
3	CY-MS1%	Yes	Yes	Yes	Yes	0.00	0.00	0.00	0.00
4	CY-MS3%	No	No	No	No	0.00	0.00	0.00	0.00
5	PCY-CS1%	No	No	No	Yes	0.00	0.00	0.00	0.00
6	PCY-CS3%	No	No	No	No	0.00	0.00	0.00	0.00
7	PCY-MS1%	Yes	Yes	Yes	Yes	0.00	0.00	0.00	0.00
8	PCY-MS3%	No	No	No	No	0.00	0.00	0.00	0.00
9	PY-CS1%	No	No	No	Yes	0.00	0.00	0.00	0.00
10	PY-CS3%	No	No	No	No	0.00	0.00	0.00	0.00
11	PY-MS1%	Yes	Yes	Yes	Yes	0.00	0.00	0.00	0.00
12	PY-MS3%	No	No	No	No	0.00	0.00	0.00	0.00
13	CF-MS1%	Yes	Yes	Yes	Yes	0.00	0.00	0.00	0.00
14	CF-MS3%	No	No	No	No	0.00	0.00	0.00	0.00

**Table 7:** Moisture management parameters of the control/bleached-only and finished samples. The values are the average of 10 measurements with a CV% less than 5%.

Sr. No.	Specimen Code	WTT (s)	WTB (s)	TAR (%/s)	BAR (%/s)	MWRT (mm)	MWRB (mm)	SST (mm/s)	SSB (mm/s)	R (-)	OMMC (-)
1	CY-control	33.25	34.14	312.11	11.20	3.12	1.24	0.31	0.15	−320.12	0.44
2	CY-CS1%	30.42	32.14	346.42	15.41	3.78	1.52	0.38	0.19	−298.24	0.68
3	CY-CS3%	31.21	33.54	327.56	13.41	3.25	1.33	0.34	0.17	−304.71	0.45
4	CY-MS1%	30.84	32.78	334.74	14.25	3.54	1.42	0.36	0.18	−300.45	0.61
5	CY-MS3%	30.48	32.41	342.81	15.28	3.68	1.51	0.37	0.19	−299.42	0.67
6	PCY-control	33.15	33.89	315.52	12.46	3.26	1.26	0.35	0.16	−322.54	0.56
7	PCY-CS1%	30.53	31.78	347.85	15.52	3.95	1.74	0.39	0.20	−297.94	0.68
8	PCY-CS3%	30.76	32.78	333.76	13.95	3.81	1.44	0.37	0.19	−301.23	0.61
9	PCY-MS1%	30.89	32.63	335.36	14.12	3.79	1.43	0.39	0.17	−312.36	0.59
10	PCY-MS3%	30.57	31.88	345.02	15.46	3.87	1.46	0.38	0.19	−300.56	0.66
11	PY-control	33.44	34.42	312.43	12.48	3.25	1.25	0.36	0.17	−319.57	0.51
12	PY-CS1%	31.16	33.08	337.47	15.45	3.84	1.38	0.40	0.19	−307.47	0.59
13	PY-CS3%	30.14	31.56	350.70	14.91	3.72	1.63	0.42	0.17	−289.73	0.66
14	PY-MS1%	30.35	31.79	335.78	18.75	3.78	1.96	0.45	1.75	−285.41	0.68
15	PY-MS3%	29.24	28.34	355.45	20.45	4.23	2.12	0.58	0.22	−254.28	0.71

## Data Availability

The original contributions presented in the study are included in the article. Further inquiries can be directed to the corresponding authors.
